# Metastatic Renal Cell Cancer to Thyroid Diagnosed by Endoscopic Ultrasound Guided Fine Needle Aspiration Technique

**DOI:** 10.1155/2017/6725297

**Published:** 2017-10-23

**Authors:** Yousef Abdel-Aziz, Tariq Hammad, Mohamad Nawras, Hayder Abdulwahid, Ali Nawras

**Affiliations:** ^1^Department of Internal Medicine, Division of Gastroenterology, University of Toledo Medical Center, Toledo, OH, USA; ^2^Department of Pathology, University of Toledo Medical Center, Toledo, OH, USA

## Abstract

Medical literature about the role of endoscopic ultrasound (EUS) in identifying thyroid lesions is limited. We present a case of secondary thyroid cancer from renal cell carcinoma (RCC) metastasis, diagnosed by thyroid EUS-fine needle aspiration (FNA) approach that was done for staging of esophageal adenocarcinoma, in a patient with 11-year history of complete right nephrectomy for RCC. An 81-year-old female patient underwent EUS for the evaluation of a newly discovered distal esophageal cancer. A hypoechoic, round, and well-demarcated mass that measured 26.9 mm × 21.9 mm was noticed in the right lobe thyroid gland. Therefore FNA was performed. The cytological results were consistent with metastatic RCC. In conclusion, EUS-FNA of thyroid nodule is a feasible and safe technique that can be used to evaluate any suspicious thyroid nodule. This case emphasizes the importance of carefully examining the thyroid gland during routine upper esophageal EUS examinations in the presence of history of nonthyroidal cancer.

## 1. Introduction

Medical literature about the role of endoscopic ultrasound (EUS) in identifying thyroid lesions is limited. We present a case of secondary thyroid cancer from renal cell carcinoma (RCC) metastasis, diagnosed by thyroid EUS-fine needle aspiration (FNA) approach that was done for staging of esophageal adenocarcinoma, in a patient with 11-year history of complete right nephrectomy for RCC.

## 2. Case Presentation and Technique

**Figure 1:**
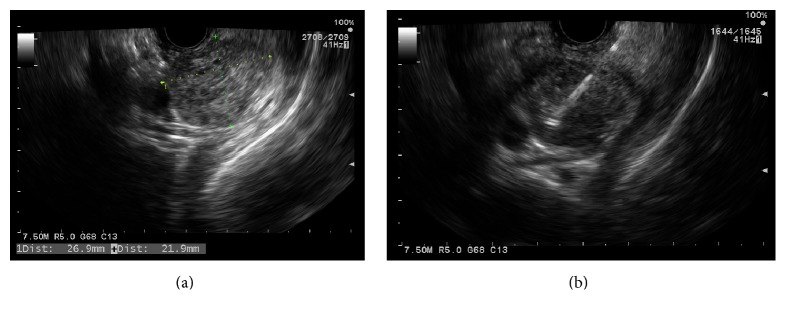
(a) EUS examination of the thyroid revealed a hypoechoic, round, and well-demarcated mass that measured 26.9 mm × 21.9 mm in the right lobe. (b) Under echoendosonographic guidance, FNA with a 25-gauge needle (Expect EUS-FNA 25 G needle; Boston Scientific, Menomonie, WI, USA) was performed successfully.

**Figure 2:**
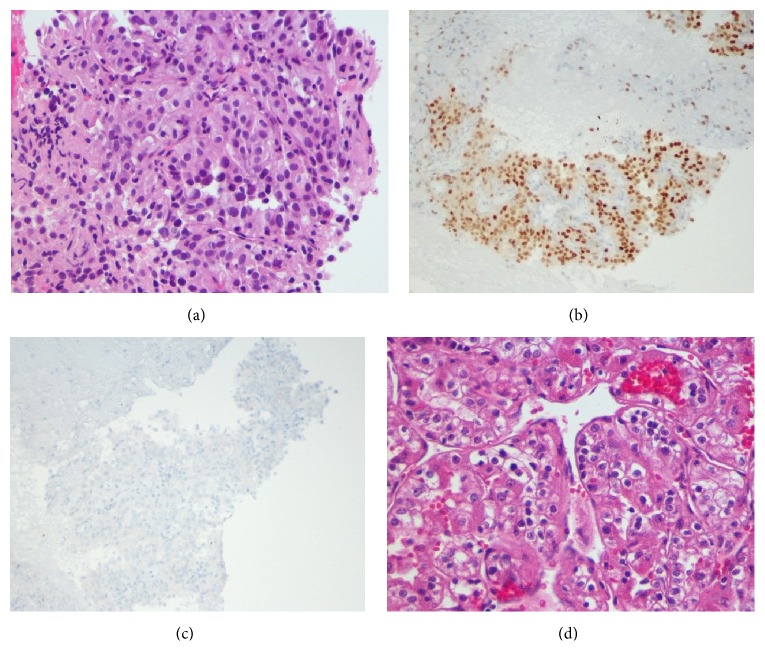
(a) Cell block of thyroid nodule FNA is displaying multiple nests of clear cells, which have clear to eosinophilic cytoplasm. These nests are separated by delicate vascular network and surrounded by lymphocytic infiltrate (hematoxylin-eosin stain, original magnification ×400). (b) Staining of cell block with PAX-8 shows strong nuclear positivity (PAX-8 immunohistochemistry stain, original magnification ×200). (c) Staining the cell block with thyroid transcription factor-1 (TTF-1) shows negative staining (TTF-1 immunohistochemistry stain, original magnification ×200). (d) Kidney section is showing multiple nests of clear cells, which have clear to eosinophilic cytoplasm. These nests are separated by delicate vascular network (hematoxylin-eosin stain, original magnification ×400).

**Figure 3:**
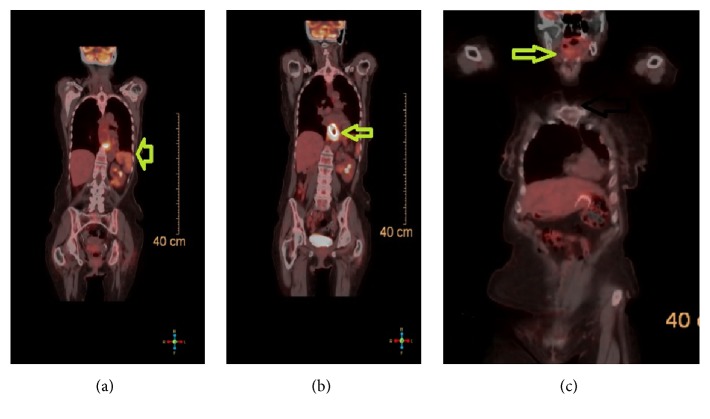
*PET scan image that was done one month after the diagnosis of thyroid RCC and esophageal adenocarcinoma*. Increased PET activity is appreciated within the lower esophagus. There was a lesion in the left adrenal gland which is PET avid that could represent adrenal metastasis (arrow). (b) A stent is present in the lower esophagus (arrow) crossing the GE junction into the stomach. Increased PET activity is appreciated within the lower esophagus, surrounding the esophageal stent. (c) Enlarging lesion in the right lobe of the thyroid (arrow) with increased PET activity compared to the remainder of the gland.

An 81-year-old female patient presented to our facility with dysphagia to both solids and liquids. For the evaluation of her dysphagia, she underwent esophagogastroduodenoscopy (EGD). Initially, EGD examination of the esophagus revealed a distal esophageal mass of 5 cm in length extending 2 cm inside the gastric cardia, that was biopsied, and the histopathologic results revealed esophageal adenocarcinoma. For staging, the patient underwent computed tomography (CT) of the chest, which showed the lower esophageal mass with mediastinal lymphadenopathy. Next, the patient underwent upper gastrointestinal (GI) EUS for assessment of the esophageal adenocarcinoma and the mediastinal lymph nodes. The endosonographic examination of the esophageal mass was performed with the scope being positioned at the proximal border of the mass. The mass was hypoechoic and heterogeneous and involved three-quarters of the circumference of the esophageal lumen, involving all esophageal wall layers to the adventitia. The EUS-FNA examination of the mediastinal lymph nodes revealed no lymphatic spread. A diagnosis of esophageal adenocarcinoma with stage of T3N0M0 was made. Upon withdrawing the EUS scope, examination of the thyroid revealed hypoechoic, round, and well-demarcated mass that measured 26.9 mm × 21.9 mm in the right lobe (Figure 1(a)). The radial echoendoscope was then withdrawn and the Olympus video curvilinear array echoendoscope was advanced through the mouth into the proximal esophagus. Color Doppler US with a linear-array echoendoscope revealed no vascular flow within or vascular invasion by the nodule. Under echoendosonographic guidance, 2 passes of FNA with a 25-gauge needle (Expect EUS-FNA 25 G needle; Boston Scientific, Menomonie, WI, USA) were performed successfully close to the upper esophageal sphincter on the right side without adverse events (Figure 1(b)). Cytopathologist was present in the room and confirmed the adequacy of the sample. The linear echoendoscope was then withdrawn and the Olympus video GIF-180 gastroscope was advanced again into the esophagus and a 23 mm × 105 mm self-expandable partially covered metallic esophageal stent was then deployed across the stricture under fluoroscopic guidance. The patient tolerated the procedure well without complications. The thyroid FNA results showed renal cell carcinoma with positive immunohistochemical stains for PAX8 and CAIX and negative for thyroid transcription factor-1 (TTF-1), thyroglobulin, and chromogranin (Figures 2(a)–2(c)). A diagnosis of metastatic RCC was made. The patient had a history of right renal cell carcinoma (RCC) status after right nephrectomy 11 years ago (Figure 2(d)). The patient was referred for further oncology workup and management. Subsequent positron emission tomography (PET) scan is shown in Figure 3.

## 3. Discussion

Metastatic disease to the thyroid gland is rare, and adenocarcinomas from the kidney, lung, breast, and colon along with squamous cell carcinomas (mainly head and neck) represent the majority of primary sites [1]. Renal cell cancers can recur, with a recurrence rate of about 60% within 2 years, 70% within 3 years, 80% within 4 years, and most of the rest within 5 years [2]. Recurrences can, however, occur many years later, with some reported cases after 30 years [3].

The use of EUS for the diagnosis of thyroid disease was first described in 2001 by Ohshima et al. [4] followed by Koike et al. [5] in 2002, who both described the usefulness of EUS in the diagnosis of esophageal infiltration of thyroid cancer. However, neither of these studies described the use of EUS-FNA for evaluation of these malignancies. In 2004, Dewitt et al. [6] described the first EUS-FNA of the thyroid in a patient presenting with a superior mediastinal mass with a final cytopathologic diagnosis of benign nodular goiter. In 2014, Foppiani et al. [7] described RCC metastasis into a hot thyroid nodule that was diagnosed by conventional percutaneous FNA.

During an upper EUS examination, the thyroid gland is usually visualized close to the upper esophageal sphincter. However, EUS is limited in visualizing the upper portions of the thyroid glands [4, 5].

In 2015, a multi-institutional study of 62 cases of secondary thyroid cancer showed that FNA biopsy has a sensitivity and specificity of 80% and 93%, respectively [1]. In 2016, Song et al. [8] described the transcutaneous ultrasonographic features and the diagnostic role of core needle biopsy in 8 patients with metastatic RCC nodule. The ultrasonographic features of metastatic RCC showed solid, hypoechoic, noncalcified nodules in all cases. 88.9% of nodules had a well-defined smooth margin. In 100% of cases, there was increased vascularity with 55% showing extensive vascularity. In this study, one FNA (11%) was able to confirm metastatic RCC (under transcutaneous ultrasonographic guidance), whereas all six core needle biopsies confirmed metastatic RCC. Alkhatib et al. [9] described the first study that evaluated the endosonographic findings of the thyroid gland during a routine upper EUS in 100 cases. The results of the study showed thyroid lesions in 12 cases, out of which three previously undiagnosed thyroid cancers were visualized during EUS (two primary papillary thyroid cancers and one anaplastic thyroid cancer). However, there was not any described case of metastatic RCC to thyroid diagnosed by EUS-FNA approach. Transesophageal EUS-guided fine needle aspiration of thyroid lesions was feasible when the lesion was in the inferior portion of the thyroid gland, and the tip of the scope was at 18 cm or more from the incisors. EUS has the advantage of assessing esophagopharyngeal invasion by thyroid cancer which can affect the staging and operative planning [4, 5]. Additionally, the sensitivity, positive predictive value, and negative predictive value of EUS tend to be better than those of magnetic resonance imaging (MRI) and esophagography [4]. Furthermore, EUS with FNA of thyroid lesions can be considered in case transcutaneous ultrasound and FNA are either unsuccessful (e.g., in case of a large retrosternal goiter) or contraindicated (e.g., because of interposing vessels). The true incidence for adverse events of EUS-FNA is not well reported. However, the risk of dissemination could occur with both transcutaneous and EUS-FNA. Because transcutaneous ultrasound with FNA is less invasive than EUS with FNA, it should continue to be the first-line evaluation in the routine diagnosis of and obtaining cytological specimens from thyroid gland lesions. However, in patients undergoing upper endoscopy or EUS for other indications, our case illustrated that EUS-FNA of thyroid lesions found during upper gastrointestinal EUS is feasible and safe.

## References

[1] Pusztaszeri M., Wang H., Cibas E. S. (2015). Fine-needle aspiration biopsy of secondary neoplasms of the thyroid gland: A multi-institutional study of 62 cases. *Cancer Cytopathology*.

[2] McNichols D. W., Segura J. W., DeWeerd J. H. (1981). Renal Cell Carcinoma: Long-Term Survival and Late Recurrence. *The Journal of Urology*.

[3] Yadav S., Bennani-Baiti N. (2014). Renal cell carcinoma metastasis to the thyroid: How long is long enough?. *Acta Oncologica*.

[4] Ohshima A., Yamashita H., Noguchi S. (2001). Usefulness of endoscopic ultrasonography (EUS) in diagnosing esophageal infiltration of thyroid cancer. *Journal of Endocrinological Investigation*.

[5] Koike E., Yamashita H., Noguchi S. (2002). Endoscopic ultrasonography in patients with thyroid cancer: Its usefulness and limitations for evaluating esophagopharyngeal invasion. *Endoscopy*.

[6] Dewitt J., Youssef W., LeBlanc J. (2004). EUS-guided FNA of a thyroid mass. *Gastrointestinal Endoscopy*.

[7] Foppiani L., Massollo M., Del Monte P., Bandelloni R., Arlandini A., Piccardo A. (2015). Late-Onset Metastasis of Renal Cell Carcinoma into a Hot Thyroid Nodule: An Uncommon Finding Not to Be Overlooked. *Case Reports in Endocrinology*.

[8] Song O. K., Koo J. S., Kwak J. Y., Moon H. J., Yoon J. H., Kim E. (2016). Metastatic renal cell carcinoma in the thyroid gland: ultrasonographic features and the diagnostic role of core needle biopsy. *Ultrasonography*.

[9] Alkhatib A. A., Mahayni A. A., Chawki G. R., Yoder L., Elkhatib F. A., Al-Haddad M. (2016). Endosonographic examination of thyroid gland among patients with nonthyroid cancers. *Endoscopic Ultrasound*.

